# An empirical analysis of sport for mental health from the perspective of a factor analysis approach

**DOI:** 10.3389/fpsyg.2022.960255

**Published:** 2022-08-01

**Authors:** Lan Zhou, Sang-Ho Lee, Youshen Cao

**Affiliations:** ^1^Department of Physical Education, Northwest University, Xi'an, China; ^2^College of Arts and Sports, Dong-A University, Busan, South Korea

**Keywords:** factor analysis, physical education and sports, mental health, psychology and body, students' mental health

## Abstract

Mental health is a kind of emotional state, a good psychological state can have a positive impact on a person, physical exercise can have a positive impact on the psychological state of college students, prevent the generation of negative emotions, improve the bad emotional state, and then promote the mental health of college students. Health is an inevitable requirement to promote the all-round development of people and a basic condition for economic and social development. Health education should be incorporated into the national education system to promote the national health of the people through sports. Young people are the main force and backbone of national and social development. In order to realize the Chinese dream of great rejuvenation, we must attach importance to the development of young people and the physical and mental health of young people. In the process of compulsory education, middle school and high school period is a key stage in the gradual formation and development of students' psychology and body, but due to the large audience of China's education, the competition is more intense, which inevitably causes a lot of students to focus on exam-oriented education and neglect physical health, especially in recent years, the mental health issues of increasing concern. Through the research situation of mental health in China and the concept of mental health quality, this paper analyzes the problems of sports and mental health, and puts forward some corresponding suggestions for the problems, which has reference significance for promoting students' mental health.

## Introduction

At present, with the rapid development of science and technology, the rapid progress of society, the demand for talent is growing, through the college entrance examination, the baton shunt, become a watershed between talent flow, middle school students face examination choice, academic pressure and the main contradiction between the physical and mental health, how on the premise of easing the pressure on students, promote students' physical and mental health become one of the main problems at this stage. Faced with these pressures, it brings a great negative effect on the life and growth of secondary school students, who are not physically healthy enough and become psychologically fragile, which affects their normal life and school learning, and eventually leads to poor learning ability and a continuous low quality of life. According to the current international common understanding, the so-called health is not only physical health, but also includes psychological, moral, having good sociability and communication skills in social life, etc., which all belong to the category of health (Song, 2021). Therefore, the adjustment of physical and psychological health of secondary school students is taken as a research direction and an attempt is made to explore the way in which sports can adjust and promote the development of physical and mental health of secondary school students (Wang, 2021).

The issue of mental health has only been taken seriously in recent years and has been recognized as an important component no less than physical health (Zhang, 2021). Mental health is, in a way, more important than physical health, as it is a matter of the environment in which a person lives and whether it affects the harmony and stability of the surrounding society, and is an important topic in all aspects of family, work and life. Especially as secondary school students who are under great academic pressure, their mental health is becoming more and more important (Gou, 2021). However, the characteristics of sports to meet the psychological needs of college students. Long-term engaged in sports can effectively relieve the psychological pressure from study, interpersonal life, emotion and other aspects of college life, enhance the social adaptability of college students, and cultivate their self-esteem and self-confidence. Sports is also very suitable for college students to improve their emotions and release them. Exercise in the rhythm and melody of the exercise, the state of tension and fatigue is adjusted.

**Figure 1:**
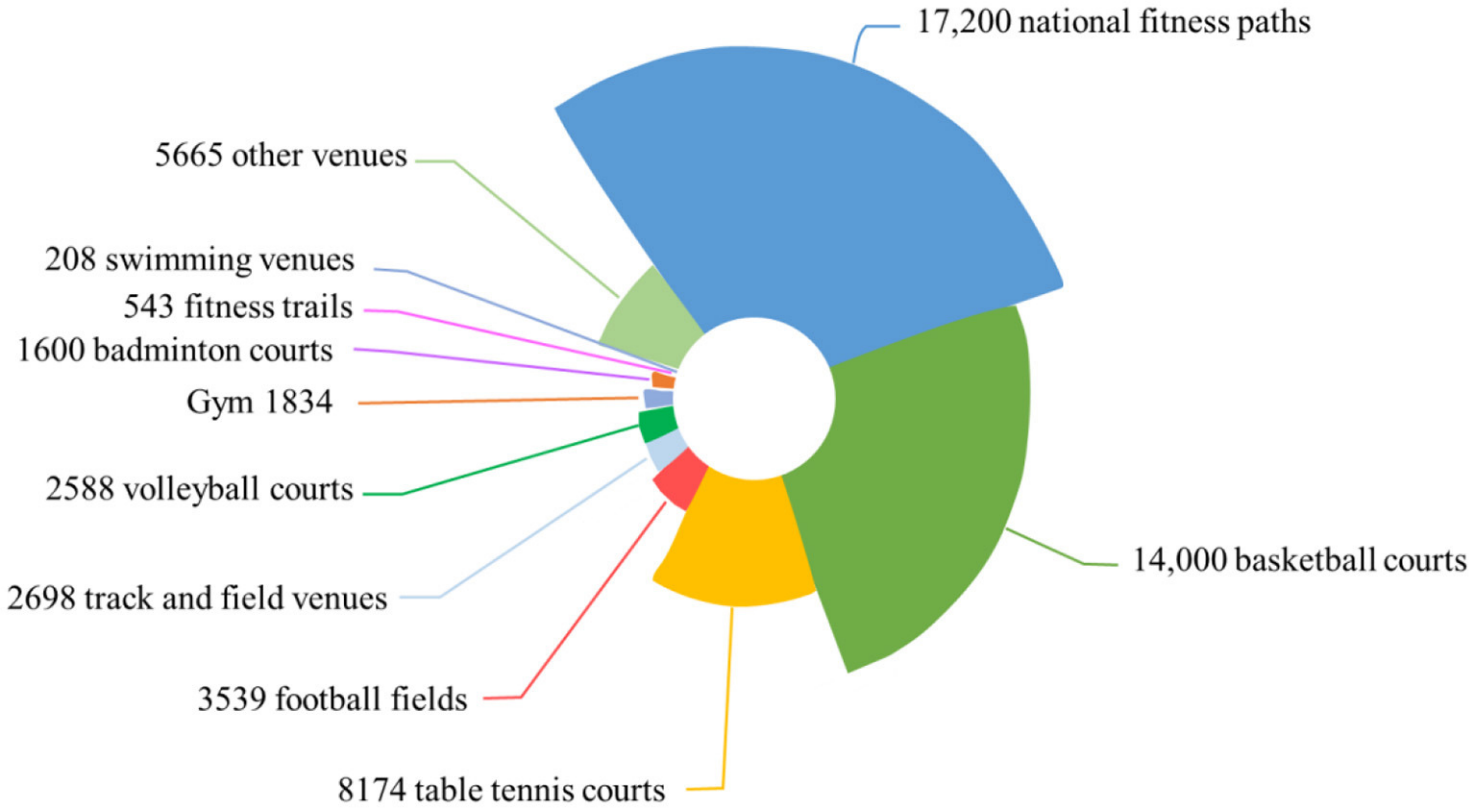
Sports field.

**Figure 2:**
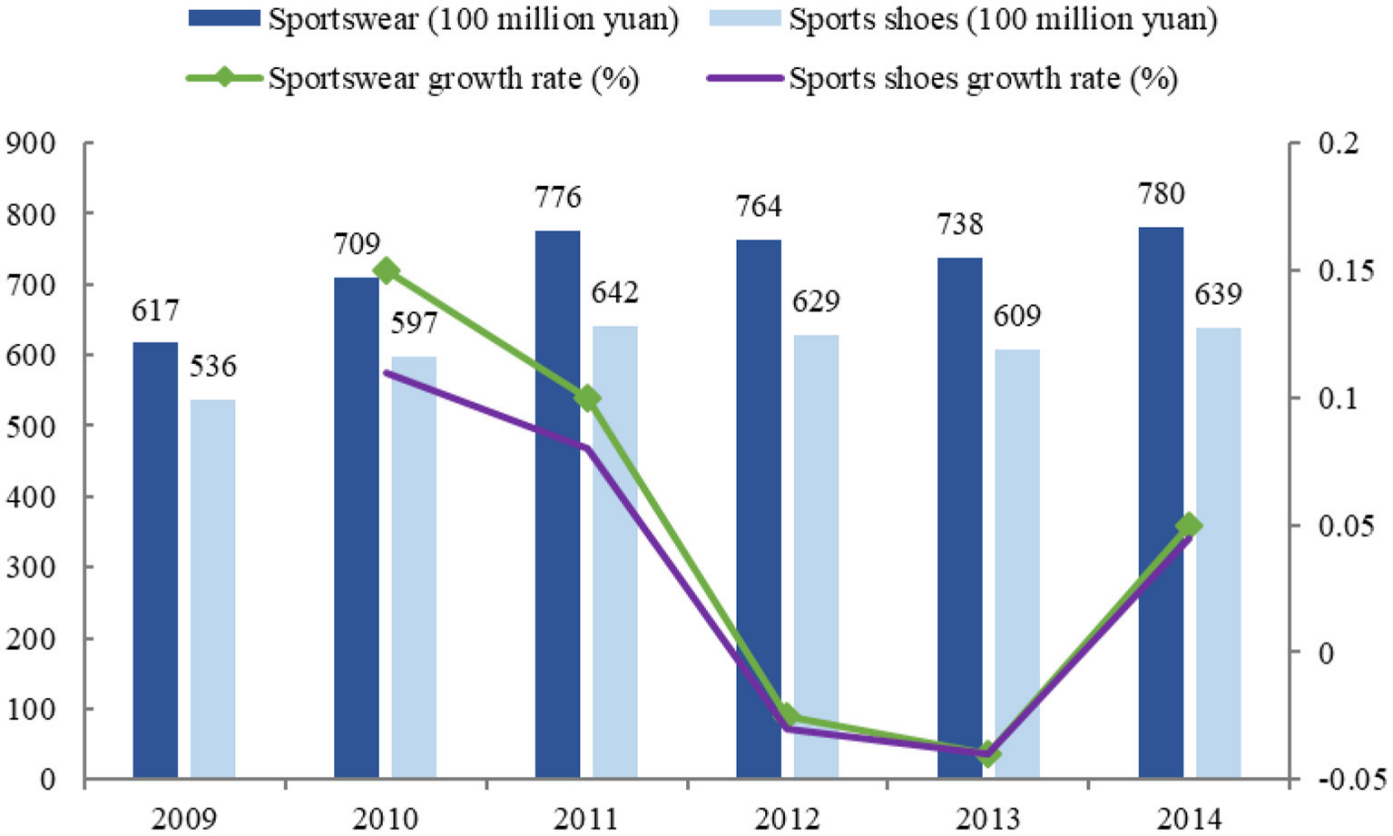
Sports equipment.

The investment in sports facilities has become more abundant, and the sale of sports products has gradually increased, as detailed in Figures 1, 2.

**Figure 3:**
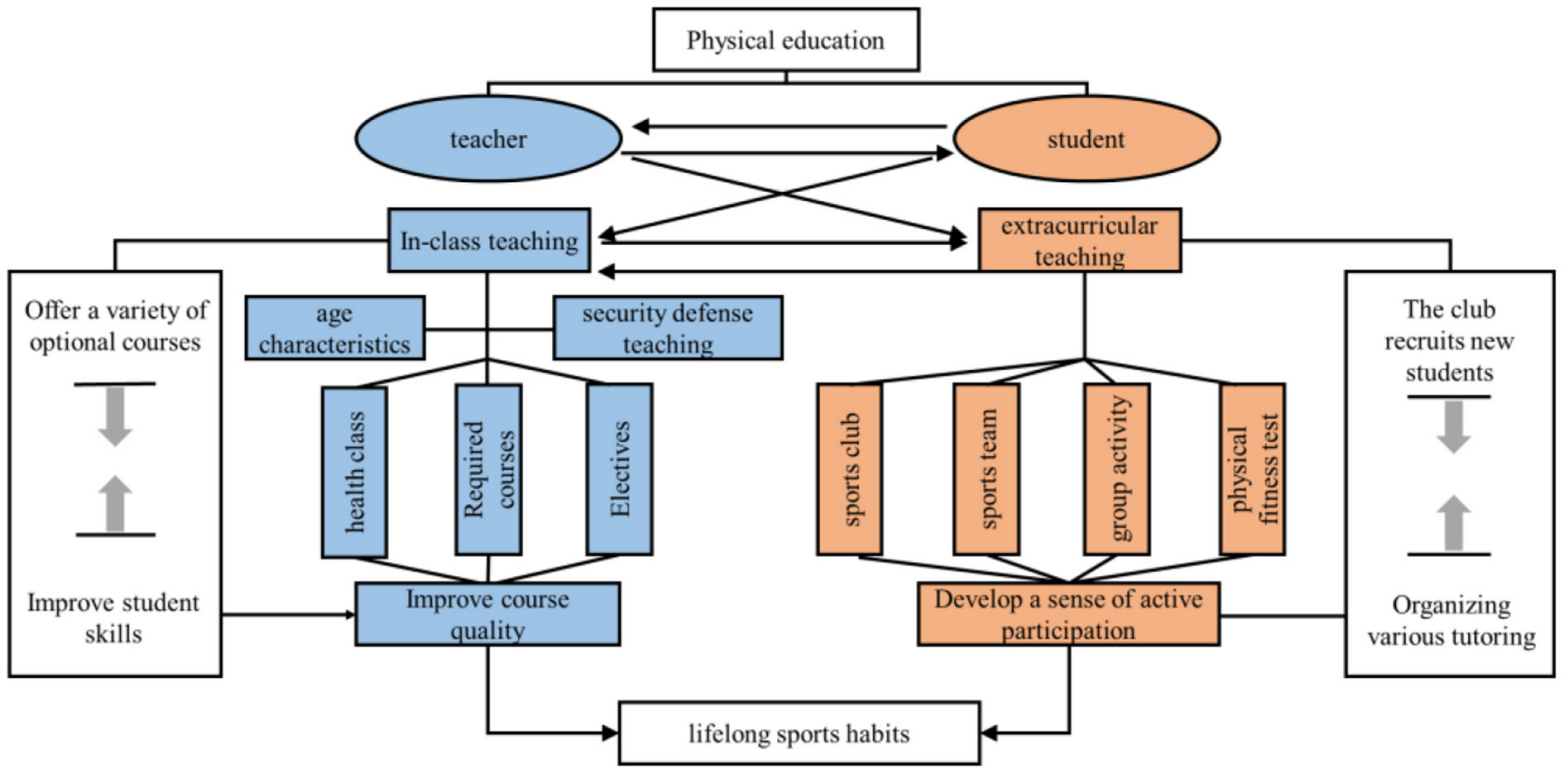
The lifelong physical education teaching mode runs through it.

The fact that sports have such rich results in promoting mental health quality is attributed to two reasons: firstly, there is still great potential for research on the psychological effects of sports on human body as an effective means of health; secondly, the issues related to mental health quality are still a hot spot worthy of in-depth research, although most of the opinions think that mental health quality contains contents inseparable from the four aspects of Although most of the opinions think that the content of mental health quality is inseparable from the four aspects of “knowledge, emotion, intention and action”, as scholars go deeper, new connotations are constantly incorporated, and some contents are also questioned, so the specific content of mental health quality of college students is still inconclusive (Wang and Wu, 2021). On the basis of existing studies, it was found that such studies suffer from two deficiencies: first, mental health benefits are considered as a direct result of physical activity, with less attention paid to the influence of dummy additional variables and the control of additional variables; second, studies of mental health quality are mostly localized, focusing on examining the positive effects of physical activity on emotions, while considering the results of emotional responses unilaterally as the structure of mental health quality (Brown et al., 2020). Secondly, studies on mental health quality have focused on examining the positive effects of physical activity on emotions from a local perspective, while considering emotional response outcomes as the main dimension in the structure of mental health quality, while ignoring the theoretical structure and mechanisms of action of physical activity for mental health quality (Bhasin et al., 2021). Therefore, this paper studies the current situation and the concept of mental health quality, analyzes the problems of sports and mental health, and does the work of promoting students' mental health.In view of this phenomenon, it is urgent to build a lifelong physical education teaching mode (as shown in Figure 3), through the interaction between teachers and students, comprehensive in-class teaching and extracurricular teaching, to carry out various sports activities in and out of class, so as to cultivate students' lifelong sports habits.

## Relevant theoretical basis

### The current situation of domestic research

At present, although China has made certain achievements in promoting sports at the secondary school level, there are still various problems and potential drawbacks due to the development of the education system to a greater or lesser extent. At present, some university campuses in China cannot highlight the main position of college students in physical education teaching, but in the traditional physical education teaching mode: traditional physical education teaching is mainly teachers teach, students learn, students' passivity is more obvious, they blindly learn under the mechanical guidance of teachers. Teaching effect directly affects the effect of students 'learning, but the present some colleges and universities for physical education although poured some effort, excessive emphasis on formalism in teaching, in the long run, physical education cannot achieve effect, students' learning efficiency is low, lead to the overall decline of physical education teaching quality in colleges and universities, this phenomenon and the development of sports slogans. Through the combing and review of China's education system, it is possible to provide a more intuitive way of thinking about the promotion of sports at the secondary school level. Since the introduction of quality education in China, more and more provinces have put on the agenda how to strengthen sports at the secondary school level, advocating reforms to strengthen the physical and humanistic qualities of our young generation. However, due to the influence of the secondary and high school exams, a large part of the reform is still a formality, and it is still difficult to get secondary school students to truly integrate into sports by changing the soup (Mittmann et al., 2020). In some provinces and cities, the examinations of the secondary school examinations have been changed from hard to soft, and the tests have been changed to physical fitness tests in order to reduce the burden on students. The result is a deepening lack of attention to physical education and sport at the secondary level, which is contrary to the goals of the reform. In addition, the lack of attention to physical education and sport in schools, and the persistence of the “unspoken rule” that physical education makes way for culture classes, has led to a reduction in students' enthusiasm for physical education and sport in secondary schools, resulting in a lack of sport as a final consequence (Kyle et al., 2021).

### Current status of foreign research

**Figure 4:**
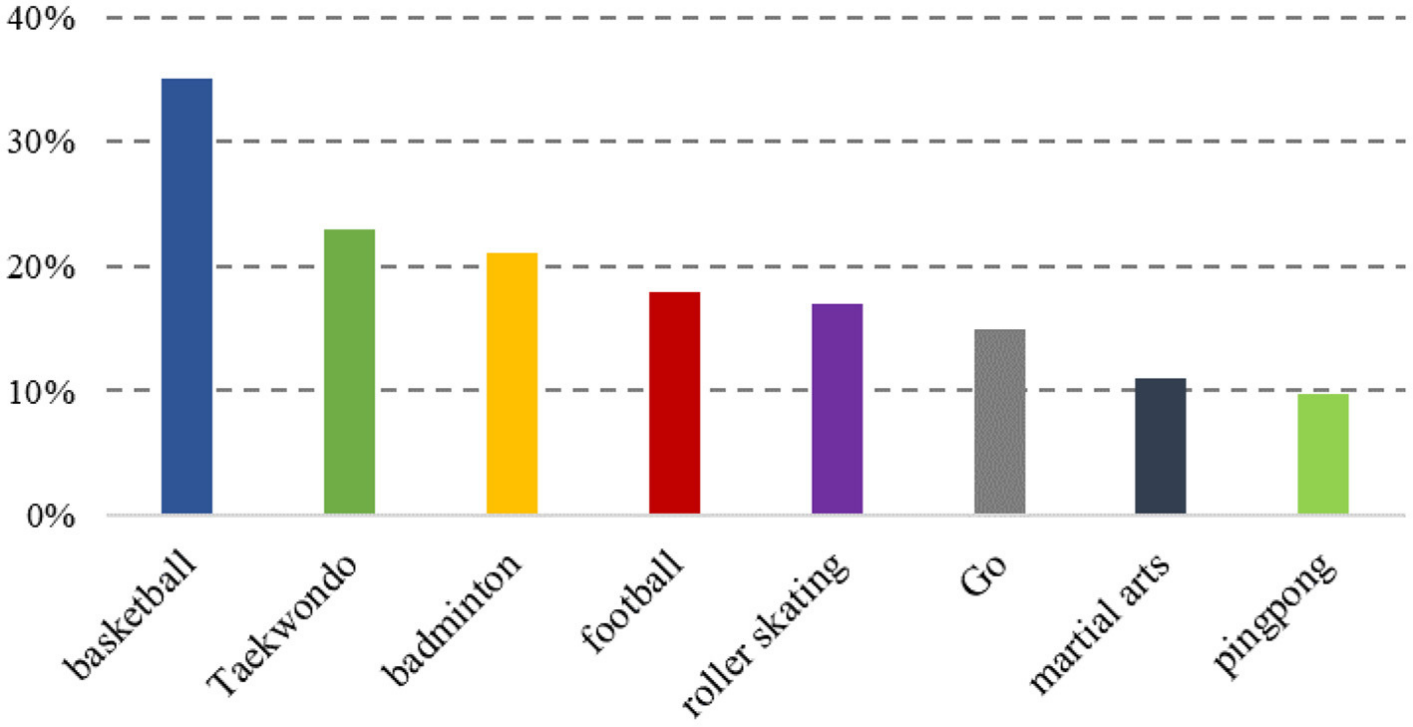
Selection of intended sports items.

The results of international scientific research in this area show that sports and activities on campus can be very good at influencing the psychological state of secondary schools and maintaining the development of students' psychological health, and that the current choice of sports by students is mainly focused on basketball and taekwondo (see Figure 4). The human body in a healthy state affects the psychological health state of individuals at certain levels, and researchers have put forward a series of hypotheses that could provide an explanation for this situation (Zhang, 2020).

#### Hypothesis of cognitive behavior

This hypothesis is based on the premise that we assume that secondary school students have the appropriate cognitive abilities, which means that when we analyze their behavior, we assume that they have positive thinking patterns and good emotional perceptions, and that they can make their own judgments and analyses of all aspects of the outside world, right and wrong. The theory of self-efficacy proposed and explained by the scholar Bantulla is similar to the theory of cognitive behavior, which believes that people have demands on their own behavior and will continue to try things that are still in progress, including the foreseeability of things. Self-efficacy is “the degree of people being confident that they can use their skills to complete a job behavior”. Bandura believes that there is an efficacy expectation. Outcome expectation refers to a person's speculation that some behavior will lead to a certain result. At the same time, he believes that when people accomplish something they find difficult, overcoming this difficulty will lead to a further increase in cognition and a further experience of self-affirmation, which will lead to the affirmation of their own value (Bidaisee et al., 2020). When this theory is applied to sports, i.e., when people overcome their bad states, such as inability to persist, feeling tired, etc., in the sports state, and thus achieve self-transcendence, then for their bad emotions that are not conducive to mental health, such as irritability, anxiety, depression, etc., are effectively discharged.

**Figure 5:**
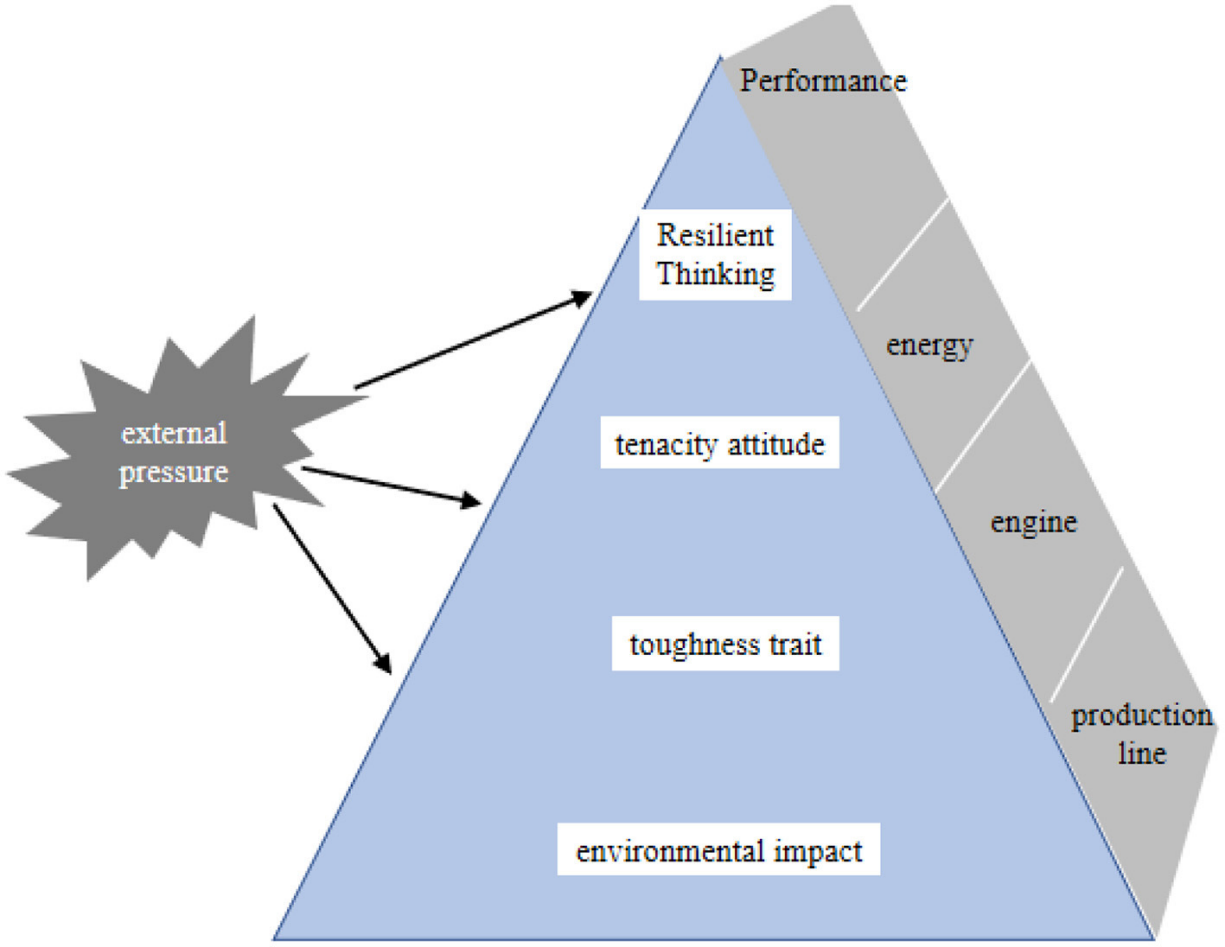
Teacher salary satisfaction level.

Based on the research prompt, Bull took cricketers' mental toughness as the research object, proposed that the mental toughness of the athletes in this sport includes five aspects, and divided the content elements of mental toughness into four levels with reference to the logic of the concept, from bottom to top, they are environmental factors, toughness traits, attitudes and thinking, thus establishing the pyramid model of mental toughness; the model emphasizes the importance of environmental factors in the development of mental toughness, and the importance of mental toughness in the development of mental toughness. The model emphasizes the importance of environmental factors in the development of mental toughness, and points out that the intrinsic pyramid model will eventually stabilize with the growth of experience, which provides important theoretical insights for cultivating and improving mental toughness (see Figure 5). From this study, we can see that students 'mental health or mental resilience is mostly determined by the environment they live in, because the environment of sports is relatively relaxed, so sports can promote students' mental health.

#### The hypothesis of distraction

This hypothesis is based on the idea that secondary school students provide some support for other behaviors in the learning process so that they can focus on other things and can make a temporary distraction from their attention, which in turn makes them depressed, anxious and other undesirable emotions decrease quickly in a short period of time (Meng et al., 2020). Sports such as jogging, brisk walking, swimming, and yoga can put students in a more natural, calm and relaxed environment, so that they can easily repeat the reinforcement without thinking too much and shift their attention through activities such as meditation, so that they can detach themselves from their previous negative emotions and enter normal thinking again. Such a shift in attention and focus can be a good way to regulate one's emotions. It has been found that if one can persist in sports for a long time, it can effectively maintain and improve one's mental health.

#### Social interaction hypothesis

To be able to interact with people around us normally, and to feel the way between people and feel happy, which can promote their own mental health, this is a premise of the social interaction hypothesis. This is a premise of the social interaction hypothesis, that is, the interaction between us and other people brings us a feeling that is not completely in a state of pleasure, there will be anxiety, sadness and other negative emotions, then it will have a negative impact on our physical and mental health. And participate in sports is a kind of social communication, because sports needs to cooperate with friends, the practice standard research, sports is let oneself in a relaxed and comfortable state of a basic and fast way, negative is almost zero, insist on exercise is not only good for physical health, for mental health.

#### Dopamine hypothesis

Some studies have shown that our neurotransmitters contain chemicals that produce certain improvements in mental health when they are secreted and transmitted between the neuromuscles and nerves. When a person's mental state is negative or not very pleasant, the secretion of these substances decreases, while when a person is in a pleasant or more relaxed state, the secretion of these substances increases, thus maintaining the physical and mental health of the person. Then, we can study from a medical point of view, it can be concluded that people participate in sports, through some physical activities can promote the secretion of such substances, and then achieve a relaxed and comfortable and physical and mental state we want, and thus play a protective role for our mental health.

#### Hypothesis of cardiovascular health

A review of medical and psychological literature shows that cardiovascular health plays a significant role in a person's mental health. Because depression, long-term stress, anxiety, anger, pessimism, and unhealthy life are associated with potentially harmful biological responses, including heart rate/arrhythmias, digestive system discomfort, increased blood pressure, and inflammation, and decreased blood flow to the heart. Studies have shown that when people play sports, the permeability and contraction of their cardiovascular system increases compared to normal, which promotes the flow of the blood circulation system, thus making the blood circulation system smooth and conducive to the maintenance of its health and the normal conduction of the nerve fiber system, which has a good effect on the maintenance of personal physical and mental health.

### The concept of mental health quality

Mental health quality is a local concept born in the process of promoting “case quality education” in China, and it is also a new research idea based on the reflection of the traditional mental health research at home and abroad. On the one hand, to some extent, the essence of mental health quality is the stripping of the healthy and positive aspects of the composition of mental quality, which is part of the mental quality that helps to form a healthy mental state; on the other hand, in the embarrassing situation that the research on mental health is often questioned and controversial, the issue of mental health standards has not reached a consensus among scholars, while the research on mental health quality derived from it has become On the other hand, the issue of mental health standards has not reached a consensus among scholars, and the research on mental health quality has become an important breakthrough and new attempt to explore mental health and quality education. To understand the concept of “mental health quality”, we need to start from quality, psychological quality and mental health, which firstly originated from psychopathological research with biological basis, but as the research progresses, the single genetic theory is gradually overturned, and it is pointed out that “quality is the genetic basis of the individual. The quality is formed through the interaction of practical and mental activities and environmental factors, and eventually internalized into relatively stable, basic and implicit qualities of the individual (Nordgreen et al., 2021). Psychological quality is a subordinate concept based on the concept of quality, which is also the core and key link of quality education. Psychological quality originates from the background of local quality education and, compared to physical quality, is proposed as a psychological quality with stable, basic and derivative characteristics that is eventually internalized through the continuous strengthening of psychological aspects when interacting with the external environment. There are differences in the understanding of psychological quality due to different research directions, and there are views that summarize psychological quality as the unity of mental health quality and intellectual quality, and emphasize that psychological quality is the guarantee for individuals to maintain a psychologically healthy state in life, study and work. The study of mental health quality is not only an expansion of the positive psychology research trend abroad, but also a new breakthrough in the study of college students' mental health in China. Mental health quality and mental health are essentially two sides of the same coin describing the phenomenon of mental health, and the concepts and criteria of both are inseparable from the analysis of the basic psychological structure of human beings. Mental health quality refers to the intrinsic and basic psychological qualities of human beings, focusing on “quality”, which is reduced to the description of psychological quality and ability, reflecting that individuals with a high level of mental health quality will have more stable psychological characteristics and are less likely to be disturbed by external factors and have psychological problems; while mental health is a state of mental Mental health is a state of mental well-being, which is characterized by sound cognitive function, normal intelligence, stable and positive emotion, sound personality, perfect will and good social adaptation, and harmonious interpersonal relationships, focusing on the description of “state”. Mental health quality is closely related to mental health state, and mental health is the ideal mental function pursued by mental health quality, which includes the basic indicators of mental health from the conceptual level, which also indirectly clarifies the relationship between mental health and mental health quality, that is, mental health is the external expression and extension of mental health quality, and mental health quality is the internal foundation and direction of mental health formation.

## Interview survey on mental health status

### Research instruments

According to the requirements of psychometrics, the quality of a questionnaire or scale is generally assessed by its reliability, which is determined by the reliability coefficient and the model fit index. +The smaller the value, the better the model fit is, and the indices of GFI, AGFI, CFI, IFI, NFI, etc. are above 0.9, which indicates the better the model fit is. The following are the results of the reliability analysis of the research instruments used in this study.

This result shows that the questionnaire has good reliability. The results of exploratory factor analysis of the data using principal component analysis and maximum variance method showed that the KMO value was 0.90 and the significance level of Barlett's sphericity test reached 0.000, indicating that the data of this study were suitable for factor analysis. The results of the principal component analysis showed that the eigenvalues of the eight items corresponding to psychological resilience were all >1, and the factor loadings ranged from 0.75 to 0.83, all of which were >0.4, with a cumulative contribution of 63.00%. The factor loadings of each question item are shown in Table 1.

**Table 1:** Factor loading diagram.

**Item**	**Factor loading**
**I believe I have the ability to achieve my goals**.	**0.75**
**When performing tasks, I can control the focus of my attention**.	**0.80**
I worked hard and persevered in overcoming difficulties.	0.83
I strive for every success.	0.83
In most cases, I can find the positive side.	0.80
I can master my emotions and express them in the way I want.	0.75
I am able to apply appropriate skills and knowledge when facing challenges.	0.80
I effectively use the knowledge and skills I need to achieve my goals.	0.79

Table 2 shows the results of descriptive statistics of college students in terms of exercise volume, mental toughness and mental health qualities, which indicate that college students are at a moderately low level of exercise volume, at a moderately low level of mental toughness and at a moderately high overall level of mental health qualities (Lavingia et al., 2020).

**Table 2:** Levels of exercise, mental toughness and mental health qualities.

	**Min**	**Max**	**M**	**SD**
Amount of exercise	0.00	100	27.51	21.53
Mental toughness	1.00	7.00	3.80	1.44
Mental health diathesis	2.15	4.49	3.38	0.32

Table 3 shows the results of the test for differences in exercise among college students in different grades, from which it can be seen that the main effect of grade on exercise was significant (*F* = 5. 06, *p* < 0. 01). Subsequent two-by-two multiple comparisons, shown in Table 4, showed that there was a significant difference between senior year and the other three grades (*P* < 0.01). This is mainly because the senior students began to be busy finding a job or postgraduate entrance examination, no time to do sports, on the other hand, the school does not require the senior students' sports, only for other grade students have sports requirements.

**Table 3:** Test for differences in exercise volume by grade.

	**Freshman**	**Sophomore**	**Junior**	**Senior**	***F***	***P***
Amount of exercise	27.19 ± 19.8	30.48 ± 22.1	29.03 ± 20.5	22.20 ± 23.1	5.06	0.00

**Table 4:** Test for differences in exercise volume by grade.

	**Freshman and sophomore (** ***t*** **)**	**Freshman and junior** **(*****t*****)**	**Freshman and senior (** ***t*** **)**	**Sophomore and junior (** ***t*** **)**	**Sophomore and senior (** ***t*** **)**	**Junior and senior** **(*****t*****)**
Amount of exercise	−3.29	−1.84	4.99*	1.45	8.28**	6.93**

* and ** respectively represent that the corresponding coefficient statistics are significant at the level of 10% and 5%.

## Suggestions for enhancing students' mental health

### School sports should be implemented in practice

The high academic demands of students and the unsatisfying after-school life make it difficult for them to have the energy for sports, and the lack of supervision of academic level tests makes students hold a relaxed state for physical education and give up sports, which leads to the weakening of their physical fitness in the process of self-indulgence. This objective factor, based on the traditional examinations in our country, makes the teaching format of physical education still more traditional and unchanged (Semlyen and Ellis, 2020; Salkovskis, 2021; Alanna et al., 2022). Schools need to implement physical exercise attendance. Students are not allowed to be absent, late or leave early when participating in various physical exercise activities organized by the school. Students who cannot ask for leave in advance; those who are absent without reason shall be considered absent, and those who leave the school shall be dismissed early. And seriously implement the “two exercises”, “two lessons” routine. When ringing the preparatory bell, the PE committee shall be responsible for counting and counting the number and making written records; after ringing the bell, report the number to the PE teacher; meanwhile, strictly implement the physical exercise discipline. Students to participate in a variety of physical exercise activities, to wear appropriate, action to be quickly, exercise seriously, discipline to be strict.

Mental health quality is a positive psychological quality inspired by the development of positive psychology. For the college students who have frequent psychological problems nowadays, the development of mental health quality can, to a certain extent, influence the physiological, psychological and social functions of college students and promote the better adaptation of college students to the development of society. Based on the results of the previous study, it is clear that the mental health quality of college students is significantly correlated with the level of sports and mental toughness, therefore, the development and improvement of mental health quality of college students can actively intervene in the mental quality of college students in Korakan education. Therefore, the first step to improve the mental health of college students is to improve their perceptions and attitudes in multiple ways and strategies, and to develop a school-based culture with special features, such as campus and classroom culture and activities. Colleges and universities can create school-based cultures with their own characteristics of mental health quality, and different disciplines or classes can also carry out special activities according to the characteristics of their disciplines to create a positive and healthy cultural atmosphere, so as to cultivate proper cognition of mental health quality among college students and encourage students to know themselves objectively and be happy with themselves. Secondly, college students should manage their emotions appropriately and maintain positive and optimistic emotions. Positive emotions can infect the students around them, and it is necessary to properly express and transmit positive emotions, as well as to regulate and control negative emotions at the right time. Again, university is an important stage to cultivate students' sound personality, and the main task of university education is to teach students how to behave, which means to cultivate students' independence and integrity of personality, and the independence of personality will motivate students to maintain a more positive attitude toward people. As Confucius said, “I have to reflect on myself three times a day. Always reflect on your own shortcomings, correct your shortcomings and discover your own strengths, improve your motivation to achieve, cultivate your sense of success, and gradually improve your will quality under the impetus of your goals. Finally, students are encouraged to actively participate in group activities to improve their interpersonal skills. Collective activities, in which everyone is working toward a common goal, whether it is winning or innovating, can make the collective environment more dynamic, and this dynamism makes the whole group more cohesive and centripetal, and also promotes the interaction between the members of the group and enhances students' interpersonal skills. Through the combined efforts of all aspects of the content, ultimately in order to promote the development and improvement of the quality of mental health of college students (Deluca et al., 2021).

### Enhancing the diversity of physical education and sports through the leverage of examination

At present, the physical education examination in Wuhan focuses on the physical quality of students, and a comprehensive score is given through the combination of the usual results and the examination results. In the assessment items, the items set can indeed make a more scientific assessment of students' physical quality to a certain extent, but the relevant items lack a certain degree of sport, which cannot attract students to promote physical and mental health development through sports in the after-school period. In some cities in China, when physical education is included in the secondary school examinations, a combination of compulsory and optional items is adopted, so that students, in addition to passively completing the physical education items required by the school, can also take the initiative to select subjects that they are relatively interested in and like to train, and gradually change from passive to active, their independent initiative is fully mobilized, so that students do The purpose of physical education is not only to improve their performance in exams, but also to improve their physical function and quality.

### Establishing the guiding ideology of “health first”

The guiding ideology of “Health First” is proposed in the “Physical Education and Health Curriculum Standards” formulated by the Ministry of Education, and this ideology makes all staff members pay attention to physical education, whose ultimate role is not only for academic exams but also for the physical and mental health of students, and should avoid taking up classes. It is important to avoid the idea that physical education is a sub-course or that it is not important, and to design and teach methods and techniques that take into account the curriculum standards, the students' own situation, and the school's hardware and facilities, so that students do not fall behind in physical education, but can also achieve all-round development through reasonable and effective sports that promote both mental and physical health (Abdullah et al., 2021). From the perspective of schools, the cultivation of teachers in this field can be increased, and more people can be engaged in this industry with relevant policies or supportive policies. At the same time, talents in the professional field can also avoid students due to substandard sports movements, or excessive injuries, resulting in losing interest in sports.

Students can be organized to receive frustration education courses or start experiential training outside of teaching and classroom, in which they are guided to cope with optimism and positive attitude in this experiential life, and through experiencing frustrating life, students' perseverance and self-confidence are enhanced, and they are motivated to cope positively when they encounter frustration or challenges, so as to improve their mental toughness (Iwahori et al., 2022). Through various measures and strategies, college students can gradually improve their own mental toughness level through subtlety.

## Conclusion

The characteristics of sports can meet the psychological needs of college students. Long-term engaged in sports can effectively alleviate the university life from learning, interpersonal, emotional psychological pressure, sports is conducive to improve their mental health level, sports is very suitable for college students to improve mood, release emotions, sports is conducive to college students to improve interpersonal relationship. Sports provides a special platform for everyone's communication and communication. Physical education in colleges and universities should give full play to the unique attributes of sports, actively guide college students to adjust their psychological state, and avoid and overcome psychological diseases. Make its mental and physical healthy growth. This paper discusses the validity of the influence of sports on college students 'mental health, the results show that college students in exercise, psychological tenacity and mental health quality described statistical results, the results show that the college students' exercise in medium low level, psychological tenacity in the moderate level, the mental health quality level average, but the exercise has significant differences in grade. This paper has important reference significance for promoting the development of students 'mental health work and improving students' mental health quality.

## Data availability statement

The original contributions presented in the study are included in the article/supplementary material, further inquiries can be directed to the corresponding author/s.

## Ethics statement

Ethical review and approval was not required for the study on human participants in accordance with the local legislation and institutional requirements. Written informed consent from the patients/ participants or patients/participants legal guardian/next of kin was not required to participate in this study in accordance with the national legislation and the institutional requirements.

## Author contributions

LZ and S-HL contributed to the writing of the manuscript and data analysis. YC supervised the work and designed the study. All authors have read and agreed the final version to be published.

## Conflict of interest

The authors declare that the research was conducted in the absence of any commercial or financial relationships that could be construed as a potential conflict of interest.

## Publisher's note

All claims expressed in this article are solely those of the authors and do not necessarily represent those of their affiliated organizations, or those of the publisher, the editors and the reviewers. Any product that may be evaluated in this article, or claim that may be made by its manufacturer, is not guaranteed or endorsed by the publisher.
